# Exploring the Toxicity of Oxytetracycline in Earthworms (*Eisenia fetida*) Based on the Integrated Biomarker Response Method

**DOI:** 10.3390/toxics12050310

**Published:** 2024-04-25

**Authors:** Haoran Zhou, Xiaoguang Jiao, Yunfei Li

**Affiliations:** 1 College of Modern Agriculture and Eco-Environment, Heilongjiang University, Harbin 150080, China; 13030012503@163.com; 2 College of Resources and Environmental Science, Northeast Agricultural University, Harbin 150030, China; s200201033@neau.edu.cn

**Keywords:** antibiotic, *Eisenia fetida*, biomarkers, oxidative stress, integrated biomarker response

## Abstract

Antibiotic contamination has become a global environmental issue of widespread concern, among which oxytetracycline contamination is very severe. In this study, earthworm (*Eisenia fetida*) was exposed to oxytetracycline to study its impact on the soil environment. The total protein (TP), catalase (CAT), superoxide dismutase (SOD), peroxidase (POD), malondialdehyde (MDA), glutathione S-transferase (GST), and glutathione peroxidase (GPX) oxidative stress indicators in earthworms were measured, and the integrated biomarker response (*IBR*) approach was used to evaluate the toxic effect of oxytetracycline on earthworms. A Technique for Order Preference by Similarity to an Ideal Solution (TOPSIS) and a path analysis model were used to explore the physiological and metabolic processes of earthworms after stress occurs. The results showed that SOD, GPX, and GST play important roles in resisting oxytetracycline stress. In addition, stress injury showed a good dose–effect relationship, and long-term stress from pollutants resulted in the most serious damage to the head tissue of earthworms. These results provide a theoretical basis for understanding the toxic effect of oxytetracycline on soil animals, monitoring the pollution status of oxytetracycline in soil, and conducting ecological security risk assessment.

## 1. Introduction

Antibiotics are either produced by bacteria, fungi, other microorganisms, and higher animals, or they occur as synthetic chemicals that can interfere with the development and functioning of some living cells [1]. The annual use of antibiotics in China is approximately 16.2 thousand tons, which is more than the sum of the respective numbers of the United States, the United Kingdom, Canada, Denmark, and other countries [2]. About 50% of surface water in the United States has antibiotics at ppb concentration levels [3,4]. Antibiotic concentrations ranging from 30.0 to 70.0 ng/L were detected in the Elbe River and its tributaries in Germany [5]; the concentration of antibiotics in the inlet and outlet water of the sewage treatment plant was as high as 7000.0 ng/L [6]. A large amount of antibiotics enters the agricultural soil ecosystem through field irrigation, surface runoff, or through sources such as sludge farming and returning animal manure to the field [7]. In soils with higher levels of organic fertilizer application, a greater accumulation of antibiotics was found [8,9]. In the soil of the Huanghuaihai Plain in China, 20 antibiotics with a total concentration level of 1.62–575.0 ng/g were discovered [10]; the content of tetracyclines detected in the soil of the vegetable base in the Pearl River Delta of China reached up to 242.6 μg/g [11].

Residual oxytetracycline in soil can migrate and accumulate within plants, thereby affecting plant quality and yield [12]. It can also affect the normal physiological activities of soil animals [13]. At an oxytetracycline concentration of 500 mg/L, it can induce DNA damage in earthworm coelom cells [14]. Oxytetracycline can also reduce the heart rate of zebrafish embryos and have adverse effects [15], such as inhibiting the growth of *Euplotes vannus* [16]. When the concentration of oxytetracycline is higher than 9.21 mg/L, it has a significant inhibitory effect on the growth of alfalfa [17]; 5 mg/kg of oxytetracycline significantly inhibited the growth of vegetables such as cucumber, lettuce, and tomato, reducing their quality [18]. It even significantly inhibits the SOD activity of Chinese cabbage and lettuce [19]. In humans, the ingestion of excessive amounts of animal-derived food with residual oxytetracycline can cause toxic reactions, damage to the stomach and liver, enamel dysplasia, microbiome disorders, allergic reactions, superinfections, and teratogenesis [20]. This compound may thus inflict extensive damage on soil ecosystems and human health [21]. So far, few studies have examined its impact on soil ecosystems; however, assessing the toxic effects of oxytetracycline on soil biota is crucial in order to identify its ecological risks to the soil ecosystem.

Earthworms are considered “soil ecosystem engineers”. They are at the bottom of the food chain and are easily exposed to various pollutants in the soil; thus, they are model organisms for environmental health research and are widely used to assess the health of agroecosystems [22,23]. However, there are numerous indicators involved in the oxidative stress effect in earthworms, which have strong complexity, and individual analysis cannot effectively evaluate the toxic effects of pollutants on organisms [24,25]. It is urgent to find suitable analytical methods for scientifically characterizing the toxic effects of pollutants.

The toxic effects of environmental pollutants can be evaluated according to biomarkers that are indicators of the sensitivity of organisms to pollutants [26]. However, sensitivity to pollutants differs between biomarkers and may change over time; thus, analyzing the response of a single biomarker to pollutant exposure may not reliably reflect overall toxicity to the organism. The comprehensive integrated biomarker response (IBR) can quantify the combined biological effects of different biological indicators, and it has been widely used in the comprehensive study of the determination of pollutant toxicity [27,28]. The IBR can intuitively reflect differences in pollutant toxicity, quantify the impact of antioxidant effects, and further accurately and effectively predict differences in toxicity [29]. In the current study experiment, oxytetracycline hydrochloride was used as the exogenous additive, and *Eisenia fetida* was used as the test organism to simulate the impact of contaminated soil on the antioxidant system of earthworms in the actual environment. Firstly, based on the comprehensive biomarker response index method (including total protein (TP), catalase (CAT), superoxide dismutase (SOD), peroxidase (POD), malondialdehyde (MDA), glutathione S transferase (GST), and glutathione peroxidase (GPX)), we evaluated the toxic effects of earthworms under oxytetracycline stress. Secondly, with the help of mathematical modeling ideas, the Technique for Order Preference by Similarity to an Ideal Solution (TOPSIS) model was combined with the path analysis model to explore the physiological metabolic processes of earthworms after stress occurs. This study provides a scientific basis and experimental basis for ecological risk assessment of polluted soil.

## 2. Materials and Methods

### 2.1. Experimental Materials

Oxytetracycline hydrochloride with 89.62% purity was purchased from LGC Labor Co., Ltd. (Augsburg, Germany), and artificial soil was prepared according to OECD standards [30]. Artificial soil was prepared using peat (bought at local gardening store, Harbin, China, dried and sieved in a 2 mm mesh), kaolin (Ai_2_O_3_·2SiO_2_·2H_2_O, analytically pure, Tianjin Kemio Chemical Reagent Co., Ltd., Tianjin, China), and quartz sand (mainly composed of SiO_2_, general mineral reagents) mixed at a ratio of 1:2:7. The deionized water was used to adjust soil moisture to 40% of the maximum water holding capacity, and soil pH was adjusted to pH 6.0–6.5 by CaCO_3_. After stirring evenly, it was used for experiment.

Earthworms (*Eisenia fetida*) were collected from the national-level ecological farm (Binxian Heyu Biotechnology Co., Ltd., Harbin, China) [31]. Healthy adult worms aged 60–65 days, with an obvious reproductive clitellum and with approximately 300–500 mg body mass, were selected for exposure tests. Before the experiments, the selected earthworms were washed using clean water, dried with filter paper, placed on wet filter paper, and then transferred to a dark environment for 24 h to allow emptying of the intestinal tract. Before the experiments, earthworms were allowed to acclimatize in the prepared artificial soil (at 20 ± 2 °C) for one week, after which healthy adult earthworms with clear clitellum and weight of (380 ± 20) mg were selected and subjected to intestinal cleansing for subsequent experiments.

### 2.2. Experimental Design

On the basis of preliminary experiments and according to previous results of tetracycline exposure at our laboratory [31], oxytetracycline solution at concentrations of 0, 0.3, 3, 30, 300 and 600 mg/kg was used to conduct oxidative stress experiments.

The stress test was carried out in a 250 mL triangular flask. Different concentrations of oxytetracycline solutions (18 mL) were prepared and sprayed onto 150 g (dw) artificial soil, and deionized water was added evenly to ensure the water content was about 40% and a final dose range of 0.036, 0.36, 3.6, 36, and 72 mg/kg.

After incubation at room temperature for 48 h, the content of oxytetracycline in the soil of the stress device and in the test earthworms was detected using high-performance liquid chromatography [32]. The results are summarized in Appendix D. Ten domesticated earthworms were placed in each stress device and covered with parafilm. The flask was then placed in a man-made incubator at 20 ± 2 °C in the absence of light, with 80% humidity. Each concentration test was performed using three replicates. Throughout the entire experiment, the survival rate of earthworms was 100%.

### 2.3. Sample Collection

Exposure was tested in a short-term experiment (sample of one earthworm per flask every day for the first ten days) and a long-term experiment (where one earthworm was sampled from each flask on the 10th, 20th, and 30th day). When sampling, we removed the sealed parafilm. We tilted the triangular flask 45 degrees to disperse the test soil due to the slope, exposing the worms cultivated inside. We carefully and randomly removed one worm using sterilized medical tweezers. Finally, we returned the triangular flask to its vertical position and sealed it again with parafilm. Each earthworm was washed with deionized water, dried with filter paper, and placed in a −80 °C freezer. Due to the heterogeneity of the tissue distribution of foreign pollutants in earthworms, the worms were sectioned for separate examination of head (with banding) and tail (without banding) tissue, with banding used as the boundary. Head and tail tissue of each earthworm were, respectively, placed in a glass homogenizer, and phosphate-buffered saline (pH 7.3) was added at nine times the weight of the earthworm (accurate to 0.0001 g), followed by grinding. The homogenization solution was placed in a centrifuge tube after complete grinding and was centrifuged at 4 °C and 3500 rpm for 30 min. The supernatant was then stored at −20 °C before use (storage time not exceeding 20 days).

### 2.4. Determination of Enzyme Content and Activity

The frozen supernatant was used to determine total protein (TP) content [33], activities of superoxide dismutase (SOD) [34], peroxidase (POD) [35], catalase (CAT) [36], glutathione peroxidase (GPX) [37], and glutathione-S-transferase (GST) [38], as well as the content of glutathione (GSH) [39] and malondialdehyde (MDA) [40]. The respective test kits were purchased from the Nanjing Jiancheng Bioengineering Research Institute (Nanjing, China).

### 2.5. Biomarker Response Index (BRI)

The *BRI* [41] was calculated as follows: the alteration level (*AL*) of the biomarker response was calculated by allocating (1) four points for slight changes (*AL* < 20%), three points for moderate changes (20% ≤ *AL* < 50%), two points for major changes (50% ≤ *AL* < 100%), and one point for severe changes (*AL* ≥ 100%). The following equation was applied:

AL=|BRt−BRc|/BRc

where *BRt* and *BRc* are the biomarker responses of the test group and the control group, respectively (i.e., the average value of replicates).

At the same time, according to the importance of biomarkers, a corresponding weight (W) was assigned. According to previous studies [41,42], CAT, SOD, POD, and GPX were 1.0, harmful metabolites produced by MDA due to stress at 1.2, and GST at 1.5. The *BRI* was calculated according to the following equation:

$$BRI=\sum \left(S_{n}\times W_{n}\right)/\sum W_{n}$$

where *S_n_* is the score of the biomarker, and *W_n_* is its weight.

*S_n_* and *W_n_* correspond to the score and weight of corresponding biomarker n, respectively.

In addition, the stress level of earthworms can be deduced, with a *BRI* of 3.01–4.00 indicating slight stress, 2.76–3.00 indicating moderate stress, 2.51–2.75 indicating high stress, and 1.00–2.50 indicating severe stress.

### 2.6. Integrated Biomarker Response (IBR) Index

The comprehensive *IBR* [43] was calculated as follows:

(1) Homogenization:
  $$Y_{i}=\left(X_{i}-m\right)/\delta$$

*Y_i_*: The data after homogenization processing;

*X_i_*: The average value of the biomarker determination results of each treatment group;

*m*: The total average value of biomarkers in all treatment groups;

*δ*: The total standard deviation of biomarkers in all treatment groups.

$$\delta =\sqrt{\frac{1}{N}\sum_{i=1}^{N}{\left(X_{i}-m\right)}^{2}}$$

, N=6

(2) Assignment: if the biomarkers of the treatment group are activated and compared with the control group, then *Z_i_* = *Yi*; otherwise, *Z_i_* = −*Y_i_*.

The formula for calculating the score *S* of this biomarker in each treatment group was

$$S=Z_{i}+\left|Y_{\min}\right|$$

|*Y_min_*|: All processing groups are normalized to the absolute value of the minimum value.

(3) *IBR* star chart

A hexagonal star chart was produced, and the score *S* of six biomarkers in each treatment group was expressed as the length of the radiation line in the star chart. The area enclosed inside the six radiation lines was the *IBR* value; the area of the triangle enclosed by the adjacent two radiation lines was *A_i_*. Then,

$$IBR=\sum_{i=1}^{6}A_{i}$$

$$A_{i}=\frac{S_{i}}{2}\sin\alpha (S_{i}\cos\alpha +S_{i+1}\sin\alpha )$$

$$\alpha =\arctan(\frac{S_{i+1}\sin\beta }{S_{i}-S_{i+1}\cos\beta })$$

*α*: the angle between the triangles formed by two adjacent radiation lines;

*β*: the angle between adjacent radiation lines.

### 2.7. Data Analyses

Each experiment was repeated three times, and the results were expressed as the mean ± standard deviation (Appendix A). Using SPSS 13.0 software (IBM, Armonk, NY, USA), we performed statistical analysis. We performed normal distribution and homogeneity of variance tests on oxidative stress data of the head and tail tissues of earthworms under different stress durations to determine the applicability of analysis of variance. Among them, in the normal distribution test, it was assumed that the research subjects with a certain sample size *n* (3 < *n* < 50) always conform to the normal distribution. When the Shapiro–Wilk value is close to 1, and the *p*-value is significantly greater than 0.05, it is impossible to reject its hypothesis, and therefore the result conforms to a normal distribution. In the homogeneity of variance test, it is assumed that the variances of each group are equal and meet the homogeneity of variances. When the *p*-value of the Levene value (mean) is greater than 0.05, the null hypothesis cannot be rejected; that is, the data conform to homogeneity of variance. We applied univariate analyses of variance to determine the effects of stress time and concentration on enzyme activity. We used post hoc comparison (Bonferroni method) to conduct significance tests on the mean differences between the treatment groups. Origin 2018 software (OriginLab, Northampton, MA, USA) was used for statistic tests and chart processing.

#### 2.7.1. TOPSIS Model Construction

The TOPSIS model, as a commonly used analytical model in multi-objective decision analysis, is a distance-comprehensive evaluation method. This model defines a measure in the target space to measure the degree to which the target is close to a positive ideal solution and away from a negative ideal solution, thereby evaluating the degree of physiological and metabolic changes in earthworms [44,45]. The specific steps are as follows:

Construct the feature matrix using the oxidative stress effect indices of the head and tail of earthworms as raw data. Among them, *i* represents the number of indices; *j* is the stress treatment group.

$$A=\left\{\begin{array}{cccc} x_{11} & x_{12} & \cdots & x_{1j}\\ x_{21} & x_{22} & \cdots & x_{2j}\\ \cdots & \cdots & \cdots & \cdots \\ x_{i1} & x_{i2} & \cdots & x_{ij} \end{array}\right\},i=12;j=13.$$

Construct the optimal solution vector and the worst solution vector separately:

$$X^{+}=\max\left(x_{1j},x_{2j},x_{3j},\cdots ,x_{ij}\right)\begin{array}{cc} & X^{-}=\min\left(x_{1j},x_{2j},x_{3j},\cdots ,x_{ij}\right) \end{array}$$

Using the Euclidean Distance formula, calculate the distances from the standardized vectors of each indicator to the optimal and worst solutions, respectively:

$$D^{+}=\sqrt{\sum_{j=1}^{13}{\left(x_{ij}-X^{+}\right)}^{2}}\begin{array}{cc} & D^{-}=\sqrt{\sum_{j=1}^{13}{\left(x_{ij}-X^{-}\right)}^{2}} \end{array}$$

Calculate the distance between the indicator and the optimal solution, i.e., the relative closeness:

$$CI=\frac{D^{-}}{D^{+}+D^{-}}$$

Calculate the weight values of the indices to obtain the physiological metabolic level value of earthworms: *S*. Meanwhile, based on the *Q* values under different stress times, calculate EC 50. This is summarized in Appendix E.

$$S=\frac{q_{ij}}{\sum_{j=1}^{13}Q_{j}}$$

And, $w=\frac{CI_{i}}{\sum_{i=1}^{12}CI_{i}}$, $q=w_{i}\cdot A_{ij}$, $Q=\sum_{i=1}^{12}q_{ij}$.

#### 2.7.2. Path Analysis Model Construction

Path analysis, as a multivariate statistical technique, studies the relative importance of variables by decomposing the correlation between the independent and dependent variables, dividing them into direct effect, indirect effect, and total effect [46]. In this study on the physiological metabolism process of earthworms, the overall oxidative stress effect index of earthworms was set as the independent variable: *Z*. The physiological metabolism level of earthworms obtained through the TOPSIS model is the dependent variable: *S*. Build a path analysis model as follows:

$$\{\begin{array}{c} r_{11}P_{1S}+r_{12}P_{2S}+r_{13}P_{3S}+\cdots +r_{16}P_{6S}=R_{1S}\\ r_{21}P_{1S}+r_{22}P_{2S}+r_{23}P_{3S}+\cdots +r_{26}P_{6S}=R_{2S}\\ \cdots \\ r_{e1}P_{1S}+r_{e2}P_{2S}+r_{e3}P_{3S}+\cdots +r_{e6}P_{6S}=R_{eS} \end{array}$$

Among them, *r_mn_* represents the simple correlation coefficient between *z_m_* and *z_n_*; *R_mS_* represents the correlation coefficient between *z_m_* and *S*; *P_mS_* represents the direct path effect coefficient, which represents the magnitude of the direct effect of *z_m_* on *S* when other variables are fixed; and *r_mn_P_mS_* represents the indirect path effect coefficient of *z_m_* on the dependent variable *S* through other variables, such as *z_n_*.

In the path analysis model, the coefficient of determination of the independent variable to the dependent variable is

$$K_{m}^{2}=P_{mS}^{2}+2\sum P_{mS}r_{mn}P_{nS}=2r_{mS}P_{mS}-P_{mS}^{2}$$

The residual effect coefficient of the path in this model is used to determine the applicability of the path analysis model and the availability of the results. When *g* < 0.20, it is considered that the path analysis model has already included all the information of the indicators. Otherwise, variables need to be added to improve the model until its residual effect coefficient meets the requirements.

$$g=\sqrt{1-\left(P_{1S}r_{1S}+P_{2S}r_{2S}+P_{3S}r_{3S}+\cdots +P_{eS}r_{eS}\right)}$$

The variables affected by certain variables in the model are called endogenous variables, and arrows point to them in the path diagram (indirect path effect). Variables that are only affected by factors outside the model are called exogenous variables, and there are no arrows pointing to them in the path diagram. This study only analyzes endogenous variables. Meanwhile, in the path diagram, the arrow of the direct path effect points from the independent variable to the dependent variable.

## 3. Results

### 3.1. Results of Simple Statistical Analysis

According to Appendix B, after the occurrence of oxytetracycline stress, the minimum value of *Shapiro–Wilk* in the head tissue of earthworms appeared at the POD after 7 days of stress, which was 0.818; its *p-value* was 0.112, which was greater than 0.05. However, in the tail tissue of worms, the minimum *Shapiro–Wilk* value appeared in the CAT after 20 days of long-term oxytetracycline stress, which was 0.881; its *p-value* was 0.315, which was greater than 0.05. These results indicated that after stress occurs, the oxidative stress enzyme data in the worm, including the head and tail tissues, followed a normal distribution. Meanwhile, as shown in Appendix B, the *p-value* of *Levene statistics* was the smallest in the MDA after long-term stress (head tissue: −0.741; tail tissue: 0.651, with both greater than 0.05). This illustrated that all samples passed the homogeneity of variance test. Therefore, variance analysis could be conducted.

According to the results of the analysis of variance (Appendix C), the test statistic of the oxytetracycline concentration in all treatment groups was *p* < 0.001, indicating a significant difference in the effect of the stress concentration on enzyme activity in earthworms. Meanwhile, in the short-term stress group, the test statistics for CAT, SOD, GST, and GPX for exposure time were all less than 0.001, indicating significant differences in the impact of exposure time on the activity of these four enzymes under short-term oxytetracycline stress. Further, as shown in Appendix C, the effect of the stress duration on POD enzyme activity was only significant in the tail tissue of the worm (*p* < 0.001). In the long-term stress group, the effect of the exposure time on GST and GPX showed significant differences (*p* < 0.001), including in the head and tail tissues of earthworms.

### 3.2. BRI of Various Oxidative Stress Indicators

**Figure 1:**
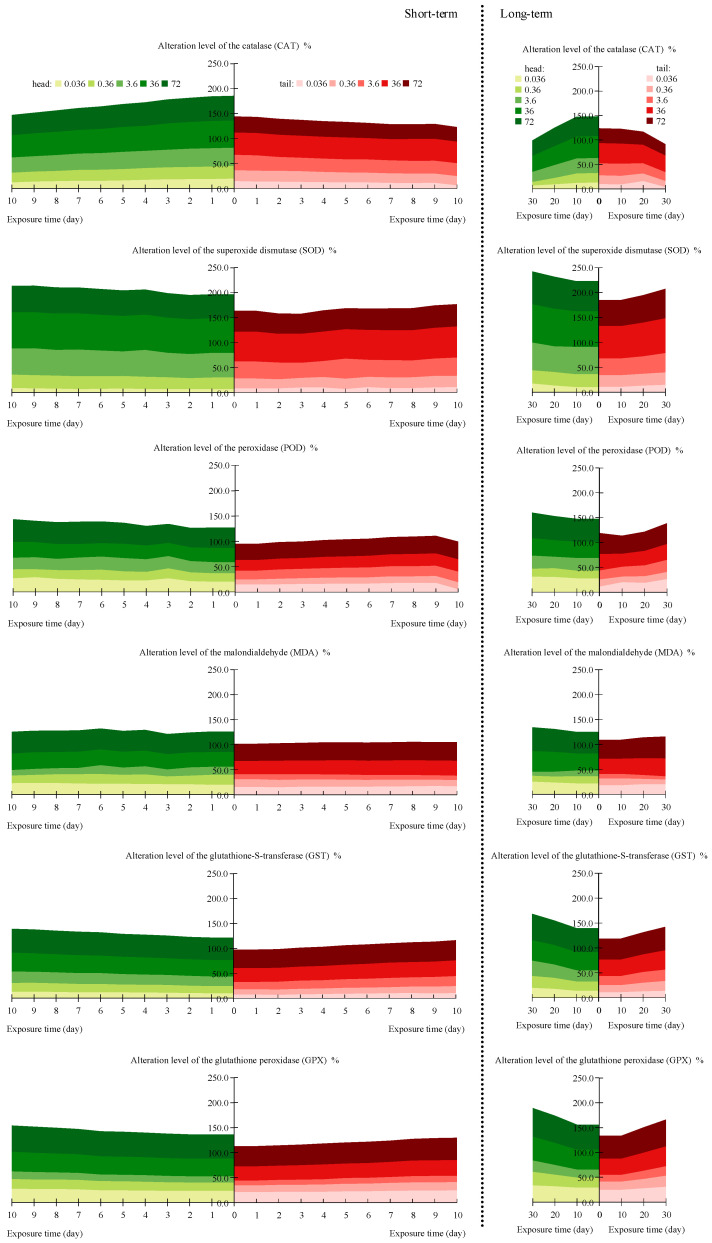
The cumulative effects of the alteration level (*AL*) of the biomarkers in earthworms after oxytetracycline stress.

With the increase in the oxytetracycline concentration, the change in CAT activity first increased and then decreased (Figure 1), and at the same exposure time, *AL* reached the maximum (0.33–0.55) when the concentration of oxytetracycline was 36 mg/kg. The change in CAT in earthworms decreased after a longer exposure duration, and *AL* reached the minimum value (0.33) on exposure day 30. The change in SOD activity showed a trend of first increasing and then decreasing with the increase in the oxytetracycline concentration (Figure 1), and AL was the highest (0.58–0.77) at 36 mg/kg; only minor changes were observed after short-term exposure, whereas a moderate upward trend occurred after long-term exposure, with the maximum value (0.77) on day 30. POD activity was lowest at 0.36 mg/kg of oxytetracycline (Figure 1) and reached the maximum at 72 mg/kg. Short-term exposure did not elicit pronounced POD activity changes, and long-term exposure resulted in a moderate increase, reaching the maximum value (0.51) on day 30. With increasing oxytetracycline concentrations, the change in the MDA content first decreased and then increased; it was lowest at 3.6 mg/kg of oxytetracycline (0.06–0.18), and at 72 mg/kg of oxytetracycline, it reached the maximum (0.34–0.47). A minor effect of the exposure time was observed. The change in GST activity showed a relatively strong dose–effect relationship (Figure 1). With the extension of the stress duration, its fluctuation decreased, and the change was the greatest (0.53) on day 30. The change in GPX activity showed a trend of first decreasing and then increasing with the increase in the oxytetracycline concentration (Figure 1). After the same stress duration, peak values appeared, respectively, at 3.6 mg/kg (*AL* was the smallest, 0.10–0.23) and 72 mg/kg (*AL* was the largest, 0.40–0.57).

**Figure 2:**
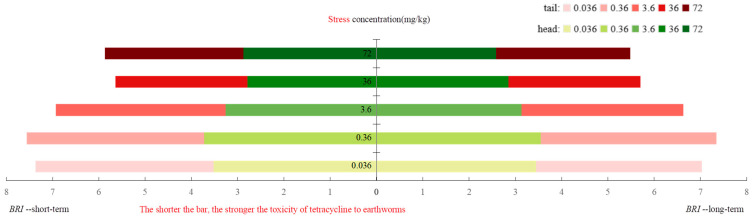
Changes of BRI of earthworms under oxytetracycline stress.

With increase in oxytetracycline concentrations, the *BRI* decreased significantly, showing a strong dose–effect relationship (Figure 2). At 36 and 72 mg/kg of oxytetracycline, the *BRI* value was the lowest in the range of 2.58–3.0, indicating moderate (2.76–3.00) and pronounced (2.51–2.75) stress, respectively. The higher the concentration of oxytetracycline, the higher the stress level in earthworms and the lower the health status. Regarding the exposure time, the *BRI* decreased more under long-term exposure to oxytetracycline. In addition, earthworm head tissue was more sensitive to oxytetracycline exposure; that is, the index of head tissue was slightly smaller than that of tail tissue. Taken together, long-term exposure to oxytetracycline exerts pronounced adverse effects on earthworm health.

### 3.3. Comprehensive Evaluation of the IBR

**Figure 3:**
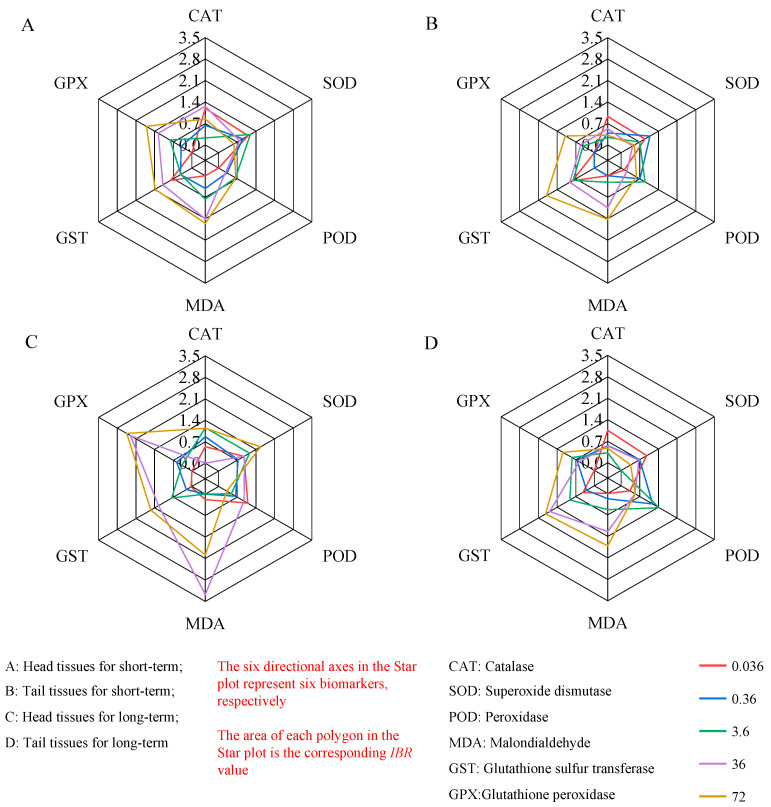
Star plot of the integrated biomarker of the earthworm under oxytetracycline stress.

**Figure 4:**
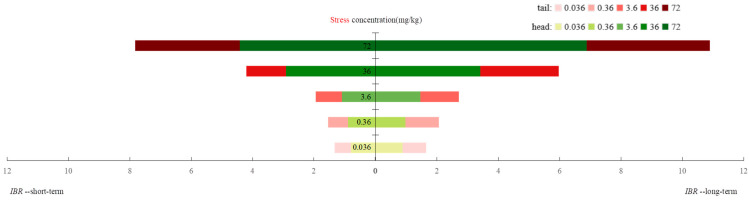
*IBR* values of earthworm head and tail tissues under OTC stress.

The high-concentration treatments (36 and 72 mg/kg) produced a large coverage area in the *IBR* star chart (Figure 3) and showed a robust dose–effect relationship, compared with the low-concentration (0.036 and 0.36 mg/kg) and medium-concentration groups (3.6 mg/kg). With regard to the exposure time, the coverage area in the star chart of earthworms under long-term exposure to oxytetracycline was larger than that after short-term exposure, showing a stable time–effect relationship (Figure 4). According to the tissue analysis of different parts of earthworms, the *IBR* value of the head tissue of earthworms was higher than that of the tail tissue.

The importance of oxidative stress indicators among comprehensive biomarkers can be inferred according to the position of each oxidative stress indicator in the star chart, and important oxidative stress indicators can be screened (Table 1). In the low-concentration group (0.036 and 0.36 mg/kg), the main oxidative stress indexes of earthworm tissues were SOD, CAT, and POD; in the medium-concentration group (3.6 mg/kg), they were SOD, POD, GPX, and GST; and in the high-concentration groups (36 and 72 mg/kg), MDA, GPX, and GST were the most important.

### 3.4. Path Analysis of Physiological Metabolism of Earthworms

According to Table 2, the minimum residual path coefficient of the 36 mg/kg oxytetracycline stress group was 0.010, indicating that the path analysis model can explain 99.0% of the original indice's information. Although the residual path coefficient of the 0.36 mg/kg stress group was relatively large, at 0.117, it still meets the requirement of a residual path coefficient of 0.20 required by the path analysis model. This indicates that the results of the path analysis model are reliable.

**Figure 5:**
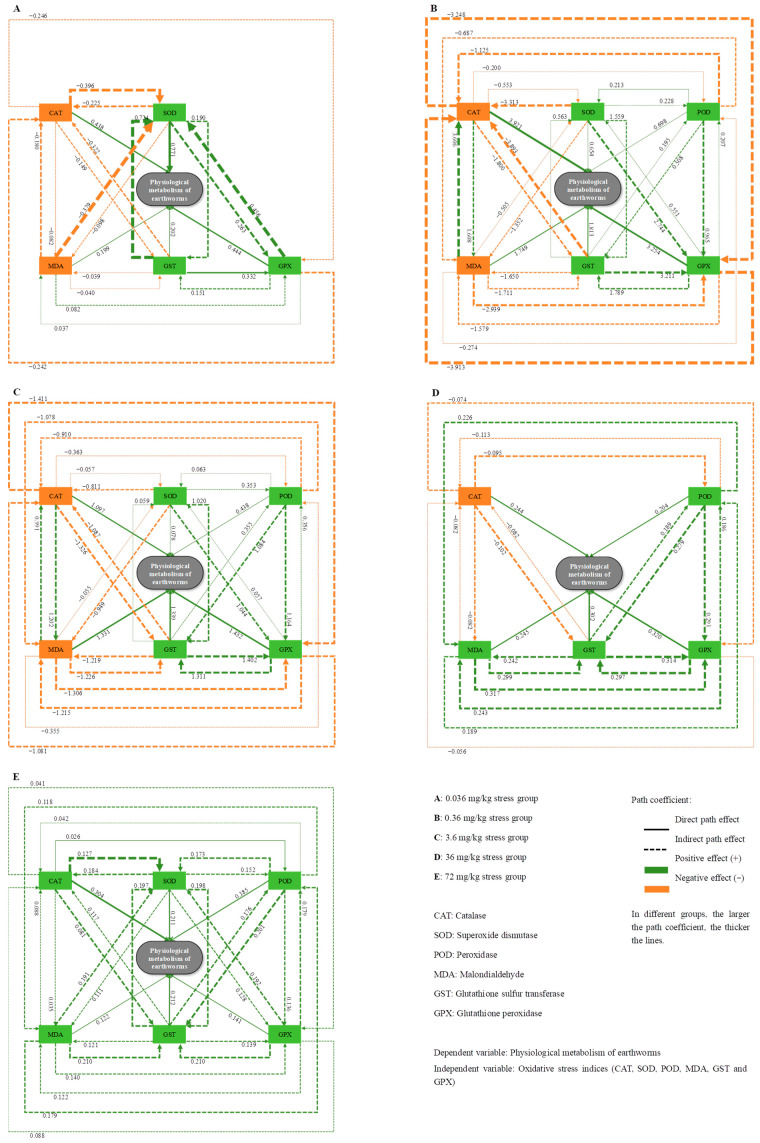
Path diagram of physiological metabolism in earthworms (the number near the line is the path coefficient).

According to the path diagram, under the stress of 0.036 mg/kg of oxytetracycline, CAT and MDA entered the model with a negative total path effect, while SOD, GST, and GPX entered the model with a positive total path effect. Among them, the total effect coefficient of SOD in earthworms can reach 0.901, which was the maximum value. From Figure 5A, it can be seen that SOD has the maximum direct path effect with a coefficient of 0.771, but its total indirect path effect coefficient was only 0.130. This was because the negative indirect effect of SOD through CAT (−0.225) offset its positive indirect effect through GPX (0.263). Although its positive indirect effect through GST was significant (0.190), its indirect path effect through MDA was minimal (−0.098).

As the stress concentration increased, all oxidative stress indices entered the path analysis model after 0.36 mg/kg and 3.6 mg/kg of oxytetracycline stress, as shown in Figure 5B,C. Both CAT and MDA showed a negative overall effect, while other oxidative stress indicators showed a positive overall effect. Among them, in the 0.36 mg/kg stress group, the maximum positive direct effect of CAT (3.921) was offset by its negative indirect effects through GST (−1.800) and GPX (−3.248), resulting in a smaller total effect (−0.271). GPX occupied the second place, with a direct path effect of 3.254, while its indirect path effect through CAT was the negative maximum value of this stress group. However, this negative indirect effect was offset by the positive indirect effect of GPX through GST, ultimately resulting in the total effect of GPX remaining positive, with a coefficient of 0.309. In the 3.6 mg/kg stress group, GPX ranked first, with a direct pathway effect of 1.432. Although the negative indirect effects of CAT and MDA partially offset GPX, GPX still maintains its maximum total effect, with a coefficient value of 0.860.

When the stress concentration of oxytetracycline increased to 36 mg/kg, only CAT entered the model with a negative total path effect, while POD, MDA, GST, and GPX entered the model with a positive total path effect. Further, GPX still had the largest positive direct effect (0.320) and total effect (0.989), playing an important role in maintaining the basic physiological metabolism of earthworms. Meanwhile, GST followed closely behind, with direct effect coefficients and total effect coefficients of 0.302 and 0.962, respectively.

As the stress concentration of oxytetracycline reached 72 mg/kg, all oxidative stress indices entered the model with a positive total effect. At this point, CAT had the largest positive direct effect (0.304), but due to its small total indirect path effect (0.310), its total effect was relatively small (0.614). SOD, with a direct path effect of 0.211 and a total indirect effect of 0.773, became the index with the highest total effect, with a total effect coefficient of 0.983. GPX and GST followed closely, with total effect values of 0.931 and 0.962, respectively. Under this stress condition, SOD, GST, and GPX played important roles in maintaining the physiological metabolism of earthworms.

## 4. Discussion

Exogenous pollutants such as antibiotics can induce oxidative stress in organisms, which plays a crucial role in the study of antibiotic toxicity [47]. In earthworms exposed to oxytetracycline in the present study, tissue biomarkers were significantly affected. However, under different stress concentrations and exposure times, complex and irregular changes may occur. Therefore, basic analysis of principal data is insufficient to evaluate such toxic effects on an organismic level [48]. In the present study, the comprehensive *BRI* method was used to evaluate the toxicity of oxytetracycline to earthworms, which can integrate complex biomarkers into a single value, minimize the risk of error, and obtain accurate toxicity and valuable risk assessment. The biomarkers refer to cellular, biochemical, physiological, behavioral, or energy changes. This can be characterized by measuring body fluids, tissues, or whole organisms, exposure to one or more pollutants, and their effects [49].

Due to the short detection cycles, high efficiency, and practicality of biomarkers, they are commonly used for monitoring and early warning of pollutants [50]. Meanwhile, the combined use of multiple biomarkers can reflect the degree of environmental pollution in a wider range [51]. Our study showed that, compared with short-term exposure, earthworms suffered more severe oxidative damage after long-term exposure, and the biomarkers showed the most pronounced changes on day 30. This is consistent with the results of previous studies [52,53].

With increasing exposure time, pollutants accumulate in earthworms; this may exceed the ability to detoxify and may cause oxidative stress damage at different levels [54]. Our dose–effect analysis showed pronounced changes in all biomarkers at 36 and 72 mg/kg. This may be because the change in antioxidant enzyme activity is related to the concentration of pollutants [55,56], and within its tolerance range, antioxidant enzyme activity increases with the increase in the pollutant concentration; however, once the tolerance threshold is exceeded, cells are severely damaged, leading to a decline in their stress capacity and antioxidant enzyme activity [57,58]. With respect to tissue-specific differences, the change level of biomarkers in the head was larger than that in the tail tissue. Meanwhile, as shown in Appendix E, the EC50 values in the head tissue were lower than those in the tail tissue. This may be because the earthworm head is more sensitive to oxytetracycline, and a previous study showed that sludge fed by earthworms increases the number, diversity, and uniformity of living microorganisms in its stomach while showing the opposite result for the posterior intestine [59]. According to Figure 1, there was a difference in the activity of oxidative stress enzymes between long-term stress for 10 days and short-term stress for 10 days. This may be due to relatively larger interference in short-term experiments [24]. On the other hand, this may be due to the influence of earthworm mucus. Research has confirmed that earthworm mucus can affect the material-cycling process in soil [60], thereby affecting the form of pollution [61] and causing differences in oxidative stress effects within the earthworm body.

According to the star chart (Figure 3), the toxicity of oxytetracycline to earthworms varied under different stress conditions, including at different stress times and concentrations. The coverage area of the treatment group with medium and high concentrations was relatively large, indicating that there were differences in toxicity at different concentrations of oxytetracycline, which also reflected the sensitivity of earthworms to stress caused by oxytetracycline in a certain concentration range. *Eisenia fetida* is known to be sensitive to tetracycline antibiotics [31]. In international detection standards, *Eisenia fetida* is considered a model organism for the detection of pollutants due to its high sensitivity [30,62]. In the current study, the *IBR* values after long-term exposure were higher than those after short-term exposure, which may be due to the accumulation of pollutants in the earthworm body increasing with the prolongation of stress time, strengthening its toxic effect and intensifying the reaction of enzyme activity to oxytetracycline stress [63].

The toxic effects of oxytetracycline may differ between tissues [27,64]. It can be seen in Figure 3 and Figure 4 that oxytetracycline had a stronger effect on the biomarkers in head tissue, and the *IBR* value of the head in the high-concentration treatments was significantly higher than that in the tail. This may be because the organs of earthworms are mainly distributed in the head tissue, and this part is where pollutants first reach. After stress occurs, neural signal transduction is blocked, causing the accumulation of a large amount of reactive oxygen species in the body, disrupting normal physiological and biochemical processes. This is similar to the findings of the author’s early research on heavy metal stress [65]. At the same time, in the process of pesticide research and development, this principle is often used to block nerve conduction, causing the biological and biochemical processes of organisms to be disrupted or disrupted, leading to their death [66,67]. On the other hand, it may be related to the microbes in the intestinal tract of earthworms. Research has found a significant correlation between the abundance of antibiotic-resistance genes in the intestine of earthworms and microbes [68,69]. Moreover, the abundant *Bacillus* and *Pseudomonas* in the intestine have the potential to degrade antibiotics in the environment [70,71]. Banerjee et al.’s study confirmed that under heavy metal stress of 25 mg/L, *Bacillus safensis*, *Bacillus flexus*, and *Staphylococcus haemolyticus* isolated from earthworm intestines can remove pollutants with a removal rate of 19.1–52.8% [72]. After 32 days of vermicomposting sludge with earthworms, the degradation rate of tetracycline antibiotics increased by 45–64% [73]. Cao et al. placed *Eisenia fetida* in soil with a concentration of 100 mg/kg of oxytetracycline for 56 days. The abundance of *Flavobacteraceae* and *Pseudomonas* increased in the soil, promoting the degradation of oxytetracycline and reducing its concentration to 33 mg/kg [74].

It can be seen from the selected main oxidative stress indicators (Table 1) that SOD, CAT, and POD play a major role in earthworms under the stress of a low concentration (0.036 and 0.36 mg/kg) of oxytetracycline. According to Figure 5A, it can be seen that after the stress of 0.036 mg/kg of oxytetracycline, SOD in earthworms plays a major direct role in maintaining their physiological metabolism. CAT, on the other hand, mainly exhibits negative indirect effects. Under stress of 0.36 mg/kg, CAT shifted toward a positive direct effect, while SOD and POD were mainly based on the total effect (as shown in Figure 5B). This may be because it is the first line of defense of the antioxidant system: after the body is subjected to external stress, SOD first catalyzes the conversion of O_2_ to H_2_O_2_, and then CAT catalyzes the conversion of H_2_O_2_ to H_2_O and O_2_ to protect cells from oxidative damage [75]; POD enhances its activity when SOD is inhibited and promotes the decomposition of H_2_O_2_ [76,77,78].

At 3.6 mg/kg of oxytetracycline, GPX and GST were selected, with the maximum positive direct effect under this stress condition (Figure 5C), confirming that they played a major role. As the second line of defense of the antioxidant system, when oxidative damage in the earthworm body increases, “detoxification” is initiated [79,80] to promote the reduction of toxic peroxides into non-toxic hydroxyl compounds and to promote the decomposition of H_2_O_2_ in the earthworm body [81,82]. Detoxifying enzymes may affect the diversity of microorganisms in earthworms [47]. When GST increases, the utilization intensity of microorganisms in earthworms to polymer carbon sources increases, and the microbial population mostly comprises microorganisms that use polymer carbon sources. An interaction between the antioxidant system and microbial community diversity was proposed, and high concentrations of pollutants may affect intestinal microorganisms through oxidative stress, thereby interfering with the nervous system [83]. MDA is a product of membrane lipid peroxidation [84], and after treatment with oxytetracycline at high concentrations (36 and 72 mg/kg) in the current study, oxidative stress was induced to produce abundant reactive oxygen species to cause lipid peroxidation, and MDA content reached the maximum, indicating that membrane lipid peroxidative damage increased and the cell membrane system was severely damaged. Excessive reactive oxygen species may cause damage to the DNA and other important macromolecules, such as protein and fat, thus further aggravating the phenomena that may elicit adverse effects, including altered gene expression, carcinogenesis, and premature aging [85,86,87]. However, we only preliminarily explored the impact of oxytetracycline on the soil ecosystem and selected a single test organism. In the future, a more comprehensive impact on a variety of soil animals should be considered.

## 5. Conclusions

Oxytetracycline stress induced oxidative damage in earthworms, and SOD, GPX, and GST played important roles in maintaining the physiological metabolism of worms.

Compared to medium and low concentrations, high concentrations caused more severe damage to the earthworm body. The toxic effect on earthworms was more pronounced under long-term stress. The head tissue of earthworms was more sensitive than the tail tissue, leading to the most severe damage to the head tissue of earthworms under long-term oxytetracycline stress.

## Figures and Tables

**Table 1:** Summary table of the main oxidative stress indicators screened under different stress times and concentrations.

Concentration Group (mg/kg)	Short-Term		Long-Term	
	Head	Tail	Head	Tail
0.036	CAT **, SOD **	CAT **, SOD **	POD **, SOD **	CAT **, SOD **
0.36	SOD **, CAT **	SOD **, POD **	CAT **, SOD **	POD **, SOD **
3.6	SOD **, GPX **	POD **, GST **	SOD **, GPX **	POD **, GST **
36	MDA **, GPX **	MDA *, GST **	MDA *, GPX **	GST **, MDA *
72	GPX **, MDA *	GST **, MDA *	GPX **, MDA *	GST **, MDA **

Abbreviations: CAT, catalase activity; SOD, superoxide dismutase activity; POD, peroxidase activity; MDA, malondialdehyde content; GST, glutathione S-transferase activity; GPX, glutathione peroxidase activity. ** indicates extremely significant at the 0.01 level; * indicates significant at the 0.05 level.

**Table 2:** Path analysis model results.

Concentration Group (mg/kg)	*p* Value	Durbin–Watson	Determination Coefficient	Residual Path Coefficient
0.036	0.0001	1.286	0.999	0.036
0.36	0.0001	2.866	0.986	0.117
3.6	0.0001	2.925	0.991	0.095
36	0.0001	2.656	1.000	0.010
72	0.0001	1.303	1.000	0.012

## Data Availability

The data that support the findings of this study are available from the corresponding author upon reasonable request.

## Appendix A. Statistical Data (*BRt* and *BRc*) of Indicators of Oxidative Stress Effects in Earthworms (Mean ± Standard Deviation)

**Groups**	**Exposure Concentration (mg/kg)**	**Exposure** **Time (day)**	**Head of Earthwom**						**Tail of Earthworm**					
			**CAT** **(U/mg prot)**	**SOD** **(U/mg prot)**	**POD** **(U/mg prot)**	**MDA** **(nmol/mg prot)**	**GST** **(U/mg prot)**	**GPX** **(U/mg prot)**	**CAT** **(U/mg prot)**	**SOD** **(U/mg prot)**	**POD** **(U/mg prot)**	**MDA** **(nmol/mg prot)**	**GST** **(U/mg prot)**	**GPX** **(U/mg prot)**
Short term	0.00	1	69.19 ± 0.068	4.59 ± 0.022	2.71 ± 0.064	0.43 ± 0.002	6.46 ± 0.045	1.86 ± 0.016	55.41 ± 0.019	3.4 ± 0.019	1.82 ± 0.018	0.24 ± 0.014	4.35 ± 0.011	2.99 ± 0.015
		2	62.62 ± 0.070	4.98 ± 0.002	2.51 ± 0.001	0.36 ± 0.004	4.76 ± 0.019	1.06 ± 0.020	55.55 ± 0.003	3.34 ± 0.006	1.42 ± 0.009	0.23 ± 0.004	7.52 ± 0.028	3.09 ± 0.023
		3	74.23 ± 0.030	3.76 ± 0.023	2.49 ± 0.038	0.35 ± 0.029	8.16 ± 0.057	1.40 ± 0.025	53.14 ± 0.060	3.39 ± 0.034	1.22 ± 0.002	0.22 ± 0.006	6.14 ± 0.039	2.73 ± 0.020
		4	72.86 ± 0.028	4.45 ± 0.043	2.46 ± 0.018	0.31 ± 0.005	8.14 ± 0.021	1.45 ± 0.029	58.92 ± 0.036	3.20 ± 0.036	1.18 ± 0.035	0.22 ± 0.028	6.27 ± 0.021	2.6 ± 0.039
		5	66.57 ± 0.083	4.38 ± 0.037	2.47 ± 0.024	0.49 ± 0.026	11.38 ± 0.004	1.49 ± 0.006	56.46 ± 0.031	3.34 ± 0.061	1.48 ± 0.015	0.21 ± 0.034	7.63 ± 0.004	2.95 ± 0.014
		6	58.68 ± 0.061	3.38 ± 0.025	2.20 ± 0.039	0.37 ± 0.039	6.12 ± 0.072	1.42 ± 0.050	59.63 ± 0.021	2.80 ± 0.036	1.13 ± 0.036	0.18 ± 0.034	7.33 ± 0.003	2.85 ± 0.020
		7	62.28 ± 0.052	3.39 ± 0.037	1.81 ± 0.038	0.52 ± 0.010	9.08 ± 0.015	2.39 ± 0.039	60.63 ± 0.038	3.31 ± 0.076	1.17 ± 0.041	0.17 ± 0.008	5.75 ± 0.012	3.15 ± 0.016
		8	76.25 ± 0.011	4.28 ± 0.001	1.77 ± 0.007	0.52 ± 0.010	9.36 ± 0.024	3.19 ± 0.026	53.39 ± 0.028	3.23 ± 0.093	1.07 ± 0.061	0.17 ± 0.037	7.90 ± 0.002	3.26 ± 0.055
		9	75.56 ± 0.002	4.21 ± 0.064	2.25 ± 0.067	0.31 ± 0.019	9.16 ± 0.002	2.41 ± 0.055	58.18 ± 0.113	3.30 ± 0.016	1.21 ± 0.034	0.15 ± 0.007	6.72 ± 0.024	2.50 ± 0.029
		10	64.95 ± 0.030	3.86 ± 0.068	2.10 ± 0.028	0.21 ± 0.016	5.19 ± 0.024	2.48 ± 0.029	59.07 ± 0.008	3.25 ± 0.058	1.35 ± 0.063	0.17 ± 0.019	6.73 ± 0.011	2.94 ± 0.015
	0.036	1	82.41 ± 0.567	4.91 ± 0.445	3.27 ± 0.164	0.52 ± 0.285	7.13 ± 0.297	2.31 ± 0.021	63.24 ± 0.214	3.7 ± 0.126	2.1 ± 0.161	0.28 ± 0.254	4.69 ± 0.256	3.62 ± 0.045
		2	74.38 ± 0.006	5.31 ± 0.047	3.05 ± 0.172	0.44 ± 0.246	5.27 ± 0.277	1.3 ± 0.016	63.31 ± 0.39	3.63 ± 0.027	1.64 ± 0.252	0.27 ± 0.353	8.08 ± 0.247	3.76 ± 0.015
		3	87.91 ± 0.311	4.02 ± 0.012	3.17 ± 0.16	0.42 ± 0.246	9.09 ± 0.268	1.73 ± 0.018	60.3 ± 0.456	3.75 ± 0.5	1.43 ± 0.228	0.25 ± 0.27	6.65 ± 0.3	3.33 ± 0.013
		4	85.54 ± 0.015	4.78 ± 0.023	3.03 ± 0.153	0.37 ± 0.249	9.08 ± 0.289	1.8 ± 0.025	66.52 ± 0.351	3.53 ± 0.065	1.38 ± 0.19	0.25 ± 0.333	6.82 ± 0.279	3.17 ± 0.013
		5	77.43 ± 0.563	4.75 ± 0.063	3.03 ± 0.161	0.6 ± 0.262	12.73 ± 0.283	1.86 ± 0.032	63.61 ± 0.005	3.59 ± 0.45	1.73 ± 0.228	0.24 ± 0.311	8.36 ± 0.286	3.62 ± 0.058
		6	67.82 ± 0.727	3.64 ± 0.14	2.73 ± 0.166	0.45 ± 0.29	6.87 ± 0.249	1.77 ± 0.02	67.08 ± 0.295	3.11 ± 0.128	1.33 ± 0.211	0.21 ± 0.306	8.05 ± 0.166	3.51 ± 0.047
		7	71.88 ± 0.578	3.63 ± 0.431	2.26 ± 0.174	0.64 ± 0.279	10.18 ± 0.255	3.02 ± 0.005	67.56 ± 0.015	3.6 ± 0.069	1.38 ± 0.191	0.2 ± 0.304	6.36 ± 0.215	3.89 ± 0.045
		8	87.48 ± 0.683	4.62 ± 0.022	2.24 ± 0.126	0.64 ± 0.204	10.54 ± 0.172	4.06 ± 0.034	59.34 ± 0.534	3.53 ± 0.444	1.26 ± 0.201	0.2 ± 0.302	8.76 ± 0.18	4.04 ± 0.037
		9	86.1 ± 0.365	4.57 ± 0.025	2.92 ± 0.138	0.38 ± 0.226	10.39 ± 0.287	3.06 ± 0.002	65.01 ± 0.721	3.66 ± 0.047	1.44 ± 0.239	0.17 ± 0.293	7.42 ± 0.244	3.11 ± 0.035
		10	72.88 ± 0.516	4.21 ± 0.442	2.68 ± 0.136	0.25 ± 0.253	5.87 ± 0.281	3.17 ± 0.006	63.04 ± 0.566	3.61 ± 0.012	1.27 ± 0.194	0.2 ± 0.303	7.46 ± 0.257	3.67 ± 0.009
	0.36	1	86.51 ± 0.379	5.66 ± 0.066	3.17 ± 0.165	0.52 ± 0.293	7.39 ± 0.255	2.17 ± 0.023	67.57 ± 0.645	4.08 ± 0.024	2 ± 0.171	0.28 ± 0.237	4.8 ± 0.292	3.39 ± 0.021
		2	78.01 ± 0.238	6.1 ± 0.492	2.95 ± 0.201	0.42 ± 0.281	5.46 ± 0.27	1.23 ± 0.031	67.68 ± 0.618	3.96 ± 0.057	1.57 ± 0.19	0.27 ± 0.225	8.33 ± 0.303	3.52 ± 0.026
		3	92.14 ± 0.42	4.67 ± 0.042	2.99 ± 0.183	0.4 ± 0.286	9.41 ± 0.293	1.64 ± 0.017	64.56 ± 0.417	4.05 ± 0.013	1.36 ± 0.166	0.25 ± 0.251	6.82 ± 0.29	3.11 ± 0.024
		4	90.1 ± 0.434	5.54 ± 0.012	2.88 ± 0.168	0.37 ± 0.289	9.42 ± 0.246	1.71 ± 0.022	71 ± 0.232	3.87 ± 0.025	1.32 ± 0.161	0.25 ± 0.232	6.98 ± 0.308	2.98 ± 0.018
		5	81.65 ± 0.377	5.48 ± 0.024	2.92 ± 0.185	0.58 ± 0.243	13.16 ± 0.224	1.77 ± 0.007	67.59 ± 0.451	4.03 ± 0.068	1.66 ± 0.17	0.24 ± 0.258	8.52 ± 0.302	3.4 ± 0.022
		6	71.78 ± 0.005	4.24 ± 0.063	2.64 ± 0.166	0.44 ± 0.317	7.14 ± 0.28	1.68 ± 0.007	71.27 ± 0.445	3.37 ± 0.148	1.27 ± 0.165	0.2 ± 0.257	8.21 ± 0.316	3.29 ± 0.013
		7	75.84 ± 0.277	4.27 ± 0.141	2.15 ± 0.149	0.62 ± 0.26	10.61 ± 0.252	2.85 ± 0.068	72.33 ± 0.388	4.03 ± 0.491	1.33 ± 0.176	0.19 ± 0.278	6.46 ± 0.29	3.65 ± 0.045
		8	91.9 ± 0.016	5.39 ± 0.491	2.08 ± 0.176	0.61 ± 0.288	10.98 ± 0.274	3.8 ± 0.085	63.53 ± 0.347	3.94 ± 0.952	1.21 ± 0.187	0.19 ± 0.274	8.92 ± 0.285	3.8 ± 0.023
		9	90.77 ± 0.542	5.32 ± 0.025	2.61 ± 0.207	0.36 ± 0.294	10.78 ± 0.31	2.87 ± 0.05	68.98 ± 0.52	4.06 ± 0.032	1.38 ± 0.179	0.17 ± 0.258	7.63 ± 0.278	2.92 ± 0.025
		10	77.6 ± 0.741	4.9 ± 0.028	2.47 ± 0.194	0.24 ± 0.295	6.12 ± 0.291	2.96 ± 0.031	70.02 ± 0.385	3.99 ± 0.514	1.54 ± 0.169	0.19 ± 0.281	7.66 ± 0.274	3.43 ± 0.009
	3.6	1	94.66 ± 0.311	6.86 ± 0.491	3.31 ± 0.158	0.5 ± 0.238	7.7 ± 0.265	2.1 ± 0.022	72.34 ± 0.218	4.56 ± 0.065	2.13 ± 0.161	0.27 ± 0.266	5 ± 0.305	3.28 ± 0.042
		2	85.55 ± 0.454	7.38 ± 0.066	3.06 ± 0.16	0.42 ± 0.28	5.7 ± 0.271	1.19 ± 0.028	70.89 ± 0.485	4.46 ± 0.413	1.67 ± 0.162	0.26 ± 0.276	8.68 ± 0.321	3.4 ± 0.037
		3	100.39 ± 0.318	5.61 ± 0.448	3.09 ± 0.138	0.4 ± 0.217	9.8 ± 0.286	1.58 ± 0.032	67.51 ± 0.528	4.42 ± 0.117	1.45 ± 0.16	0.24 ± 0.264	7.14 ± 0.285	3.01 ± 0.028
		4	98.08 ± 0.216	6.84 ± 0.133	3.08 ± 0.167	0.36 ± 0.261	9.8 ± 0.292	1.65 ± 0.014	74.64 ± 0.396	4.22 ± 0.026	1.4 ± 0.166	0.24 ± 0.237	7.3 ± 0.308	2.88 ± 0.022
		5	89.67 ± 0.362	6.54 ± 0.135	3.11 ± 0.154	0.56 ± 0.244	13.77 ± 0.293	1.69 ± 0.017	71.35 ± 0.006	4.67 ± 0.46	1.76 ± 0.16	0.23 ± 0.312	8.95 ± 0.311	3.29 ± 0.008
		6	78.1 ± 0.392	5.12 ± 0.045	2.77 ± 0.152	0.44 ± 0.243	7.45 ± 0.167	1.6 ± 0.041	75.33 ± 0.334	3.75 ± 0.062	1.35 ± 0.164	0.2 ± 0.273	8.62 ± 0.261	3.19 ± 0.006
		7	82.77 ± 0.301	5.21 ± 0.012	2.26 ± 0.191	0.59 ± 0.306	11.08 ± 0.314	2.73 ± 0.033	76.39 ± 0.017	4.45 ± 0.4	1.4 ± 0.156	0.18 ± 0.26	6.82 ± 0.305	3.52 ± 0.028
		8	100.47 ± 0.442	6.48 ± 0.023	2.17 ± 0.157	0.58 ± 0.268	11.42 ± 0.307	3.64 ± 0.045	66.97 ± 0.588	4.33 ± 0.119	1.28 ± 0.181	0.18 ± 0.282	9.36 ± 0.176	3.67 ± 0.007
		9	98.87 ± 0.379	6.47 ± 0.061	2.79 ± 0.161	0.34 ± 0.298	11.22 ± 0.305	2.77 ± 0.033	73.06 ± 0.37	4.45 ± 1.413	1.46 ± 0.171	0.16 ± 0.274	8.01 ± 0.268	2.82 ± 0.042
		10	84.83 ± 0.218	5.88 ± 0.139	2.59 ± 0.177	0.23 ± 0.293	6.37 ± 0.295	2.86 ± 0.018	74.2 ± 0.558	4.44 ± 0.045	1.63 ± 0.194	0.19 ± 0.275	8.07 ± 0.292	3.32 ± 0.017
	36	1	106.99 ± 0.184	7.78 ± 0.037	3.47 ± 0.032	0.56 ± 0.028	8.61 ± 0.316	2.52 ± 0.032	80.2 ± 0.387	5.43 ± 0.012	2.2 ± 0.16	0.31 ± 0.278	5.55 ± 0.283	3.84 ± 0.051
		2	96.08 ± 0.123	8.46 ± 0.066	3.19 ± 0.031	0.47 ± 0.031	6.34 ± 0.298	1.43 ± 0.046	80.31 ± 0.192	5.27 ± 0.023	1.72 ± 0.18	0.29 ± 0.218	9.62 ± 0.28	3.97 ± 0.032
		3	113.36 ± 0.444	6.42 ± 0.015	3.14 ± 0.032	0.46 ± 0.029	10.9 ± 0.34	1.9 ± 0.061	76.41 ± 0.364	5.36 ± 0.068	1.49 ± 0.16	0.28 ± 0.236	7.87 ± 0.298	3.52 ± 0.008
		4	109.37 ± 0.021	7.61 ± 0.161	3.13 ± 0.03	0.4 ± 0.027	10.93 ± 0.268	1.97 ± 0.033	84.69 ± 0.385	5.08 ± 0.139	1.44 ± 0.172	0.28 ± 0.25	8.06 ± 0.27	3.37 ± 0.02
		5	99.73 ± 0.012	7.51 ± 0.028	3.16 ± 0.031	0.64 ± 0.028	15.33 ± 0.267	2.04 ± 0.034	81.06 ± 0.346	5.31 ± 0.449	1.81 ± 0.16	0.27 ± 0.226	9.86 ± 0.258	3.81 ± 0.031
		6	87.18 ± 0.338	5.81 ± 0.009	2.79 ± 0.033	0.49 ± 0.029	8.27 ± 0.289	1.94 ± 0.036	85.14 ± 0.005	4.47 ± 0.861	1.39 ± 0.161	0.23 ± 0.259	9.52 ± 0.304	3.69 ± 0.011
		7	91.8 ± 0.124	5.85 ± 0.074	2.29 ± 0.03	0.69 ± 0.027	12.34 ± 0.285	3.28 ± 0.042	86.31 ± 0.286	5.29 ± 0.026	1.44 ± 0.158	0.22 ± 0.255	7.52 ± 0.242	4.09 ± 0.027
		8	111.86 ± 0.102	7.39 ± 0.078	2.29 ± 0.033	0.68 ± 0.03	12.76 ± 0.286	4.4 ± 0.025	76.73 ± 0.015	5.18 ± 0.51	1.33 ± 0.157	0.22 ± 0.265	10.32 ± 0.218	4.27 ± 0.043
		9	110.11 ± 0.319	7.25 ± 0.02	2.93 ± 0.031	0.41 ± 0.029	12.56 ± 0.301	3.34 ± 0.04	83.72 ± 0.482	5.34 ± 0.065	1.51 ± 0.18	0.19 ± 0.261	8.79 ± 0.28	3.29 ± 0.05
		10	93.89 ± 0.071	6.67 ± 0.003	2.75 ± 0.031	0.27 ± 0.029	7.16 ± 0.326	3.45 ± 0.028	84.62 ± 0.722	5.26 ± 0.455	1.68 ± 0.184	0.22 ± 0.272	8.85 ± 0.248	3.86 ± 0.026
	72	1	103.01 ± 0.383	6.79 ± 0.43	3.79 ± 0.156	0.61 ± 0.242	9.36 ± 0.299	2.77 ± 0.028	73.06 ± 0.225	4.8 ± 0.038	2.39 ± 0.187	0.32 ± 0.289	5.95 ± 0.241	4.18 ± 0.035
		2	92.81 ± 0.342	7.37 ± 0.721	3.47 ± 0.122	0.5 ± 0.211	6.88 ± 0.309	1.57 ± 0.03	73.04 ± 0.351	4.68 ± 0.189	1.87 ± 0.184	0.31 ± 0.284	10.31 ± 0.24	4.33 ± 0.06
		3	109.7 ± 0.336	5.6 ± 0.027	3.43 ± 0.148	0.49 ± 0.274	11.84 ± 0.281	2.09 ± 0.009	69.82 ± 0.321	4.74 ± 0.077	1.62 ± 0.182	0.29 ± 0.331	8.43 ± 0.277	3.84 ± 0.029
		4	107.01 ± 0.005	6.67 ± 0.044	3.41 ± 0.155	0.43 ± 0.247	11.84 ± 0.301	2.16 ± 0.033	77.03 ± 0.505	4.54 ± 0.074	1.57 ± 0.219	0.29 ± 0.327	8.64 ± 0.262	3.68 ± 0.048
		5	97 ± 0.263	6.61 ± 0.01	3.48 ± 0.151	0.69 ± 0.239	16.57 ± 0.268	2.23 ± 0.016	73.78 ± 0.547	4.72 ± 0.399	1.97 ± 0.208	0.28 ± 0.338	10.55 ± 0.25	4.18 ± 0.039
		6	84.97 ± 0.014	5.11 ± 0.022	3.13 ± 0.161	0.52 ± 0.265	8.96 ± 0.277	2.12 ± 0.011	77.57 ± 0.388	3.99 ± 0.439	1.51 ± 0.21	0.24 ± 0.327	10.15 ± 0.171	4.06 ± 0.035
		7	89.32 ± 0.491	5.14 ± 0.062	2.6 ± 0.146	0.74 ± 0.318	13.34 ± 0.291	3.6 ± 0.048	78.59 ± 0.481	4.74 ± 0.332	1.56 ± 0.219	0.23 ± 0.319	7.96 ± 0.241	4.48 ± 0.052
		8	108.71 ± 0.747	6.49 ± 0.142	2.54 ± 0.18	0.73 ± 0.287	13.76 ± 0.175	4.83 ± 0.026	68.91 ± 0.169	4.65 ± 0.549	1.43 ± 0.214	0.23 ± 0.309	11 ± 0.307	4.66 ± 0.028
		9	106.57 ± 0.5	6.43 ± 0.395	3.19 ± 0.173	0.44 ± 0.295	13.54 ± 0.304	3.66 ± 0.035	75.23 ± 0.532	4.77 ± 0.061	1.63 ± 0.193	0.2 ± 0.341	9.38 ± 0.263	3.6 ± 0.055
		10	91.11 ± 0.592	5.89 ± 0.81	3.04 ± 0.185	0.29 ± 0.256	7.65 ± 0.296	3.78 ± 0.032	76.03 ± 0.629	4.69 ± 0.502	1.82 ± 0.21	0.23 ± 0.324	9.42 ± 0.289	4.24 ± 0.066
Long-term	0.0	10	53.86 ± 0.053	3.92 ± 0.066	1.90 ± 0.026	0.23 ± 0.044	9.62 ± 0.028	1.56 ± 0.023	65.42 ± 0.014	3.56 ± 0.052	1.56 ± 0.014	0.15 ± 0.027	7.82 ± 0.004	2.7 ± 0.014
		20	52.99 ± 0.020	4.60 ± 0.067	2.27 ± 0.038	0.41 ± 0.035	10.34 ± 0.039	3.30 ± 0.02	60.60 ± 0.006	2.85 ± 0.029	1.14 ± 0.022	0.20 ± 0.003	7.67 ± 0.003	2.12 ± 0.02
		30	60.89 ± 0.043	4.17 ± 0.028	1.98 ± 0.019	0.45 ± 0.012	5.00 ± 0.021	6.67 ± 0.039	57.07 ± 0.067	3.13 ± 0.104	1.46 ± 0.038	0.21 ± 0.036	8.17 ± 0.012	2.12 ± 0.016
	0.036	10	60.44 ± 0.371	4.34 ± 0.044	2.44 ± 0.155	0.29 ± 0.292	11 ± 0.313	2.03 ± 0.023	71.48 ± 0.515	3.96 ± 0.058	1.87 ± 0.099	0.18 ± 0.241	8.73 ± 0.356	3.38 ± 0.037
		20	57.93 ± 0.166	5.23 ± 0.009	2.97 ± 0.103	0.51 ± 0.226	12.23 ± 0.268	4.36 ± 0.011	70.42 ± 0.215	3.24 ± 0.031	1.36 ± 0.111	0.24 ± 0.288	8.69 ± 0.333	2.72 ± 0.011
		30	65.07 ± 0.305	4.91 ± 0.022	2.6 ± 0.116	0.56 ± 0.212	6.01 ± 0.302	8.93 ± 0.053	59.52 ± 0.365	3.6 ± 0.5	1.85 ± 0.15	0.25 ± 0.372	9.34 ± 0.517	2.79 ± 0.02
	0.36	10	64.35 ± 0.368	4.93 ± 0.057	2.2 ± 0.143	0.27 ± 0.237	11.41 ± 0.252	1.88 ± 0.038	77.05 ± 0.012	4.41 ± 0.022	1.73 ± 0.133	0.17 ± 0.271	8.94 ± 0.253	3.15 ± 0.025
		20	59.93 ± 0.317	5.85 ± 0.12	2.67 ± 0.126	0.46 ± 0.249	12.61 ± 0.274	4.08 ± 0.032	70.1 ± 0.436	3.54 ± 0.411	1.3 ± 0.116	0.22 ± 0.288	9.01 ± 0.241	2.53 ± 0.033
		30	65.31 ± 0.005	5.28 ± 0.413	2.3 ± 0.141	0.5 ± 0.233	6.2 ± 0.26	8.5 ± 0.019	64.1 ± 0.542	3.91 ± 0.052	1.66 ± 0.137	0.22 ± 0.241	9.78 ± 0.277	2.6 ± 0.032
	3.6	10	70.35 ± 0.366	6.08 ± 0.041	2.39 ± 0.15	0.26 ± 0.353	11.8 ± 0.336	1.82 ± 0.027	81.54 ± 0.214	4.76 ± 0.027	1.86 ± 0.159	0.17 ± 0.246	9.25 ± 0.309	3.07 ± 0.011
		20	66.98 ± 0.17	6.97 ± 0.01	2.79 ± 0.098	0.45 ± 0.281	13.12 ± 0.326	3.95 ± 0.012	73.31 ± 0.389	3.85 ± 0.47	1.39 ± 0.193	0.22 ± 0.324	9.32 ± 0.467	2.47 ± 0.021
		30	73.53 ± 0.312	6.48 ± 0.02	2.5 ± 0.112	0.48 ± 0.294	6.54 ± 0.361	8.19 ± 0.059	67.34 ± 0.412	4.34 ± 0.07	1.81 ± 0.21	0.22 ± 0.298	10.05 ± 0.306	2.53 ± 0.037
	36	10	77.86 ± 0.487	6.72 ± 0.055	2.53 ± 0.136	0.31 ± 0.277	13.13 ± 0.258	2.19 ± 0.037	92.39 ± 0.363	5.88 ± 0.445	1.96 ± 0.176	0.2 ± 0.304	10.4 ± 0.256	3.58 ± 0.025
		20	73.32 ± 0.344	8.06 ± 0.115	3.02 ± 0.115	0.57 ± 0.304	14.4 ± 0.278	4.77 ± 0.038	83.48 ± 0.006	4.78 ± 0.132	1.48 ± 0.179	0.27 ± 0.287	10.39 ± 0.265	2.9 ± 0.058
		30	80.84 ± 0.005	7.38 ± 0.395	2.68 ± 0.138	0.63 ± 0.3	7.05 ± 0.309	9.86 ± 0.023	76.54 ± 0.298	5.31 ± 0.133	1.92 ± 0.166	0.28 ± 0.248	11.34 ± 0.289	2.97 ± 0.033
	72	10	75.56 ± 0.268	6.29 ± 0.021	2.77 ± 0.14	0.33 ± 0.284	14.22 ± 0.249	2.35 ± 0.037	85.01 ± 0.307	5.41 ± 0.506	2.11 ± 0.161	0.21 ± 0.278	11.09 ± 0.272	3.94 ± 0.055
		20	72.85 ± 0.011	7.53 ± 0.018	3.36 ± 0.117	0.59 ± 0.304	15.39 ± 0.311	5.09 ± 0.03	76.48 ± 0.448	4.42 ± 0.061	1.58 ± 0.179	0.28 ± 0.346	11.04 ± 0.296	3.2 ± 0.042
		30	79.91 ± 0.411	6.9 ± 0.427	2.99 ± 0.113	0.65 ± 0.343	7.64 ± 0.366	10.49 ± 0.041	69.94 ± 0.358	4.96 ± 0.431	2.06 ± 0.171	0.3 ± 0.285	12 ± 0.264	3.26 ± 0.033
Abbreviations: CAT, catalase activity; SOD, superoxide dismutase activity; POD, peroxidase activity; MDA, malondialdehyde content; GST, glutathione S-transferase activity; GPX, glutathione peroxidase activity. The control group (0.0 mg/kg) data were the *BRc*; the experimental groups' (0.036, 0.36, 3.6, 36, and 72 mg/kg) data were the *BRt*.														

## Appendix B. Assumption Test of Variance Analysis

**Groups**	**Tissue**	**Exposure Time (day)**	**Indices**											
			**CAT**		**SOD**		**POD**		**MDA**		**GST**		**GPX**	
			**Shapiro–Wilk**	**Sig.**	**Shapiro–Wilk**	**Sig.**	**Shapiro–Wilk**	**Sig.**	**Shapiro–Wilk**	**Sig.**	**Shapiro–Wilk**	**Sig.**	**Shapiro–Wilk**	**Sig.**
Short-term	Head	1	0.936	0.641	0.958	0.797	0.896	0.388	0.864	0.244	0.929	0.587	0.941	0.674
		2	0.938	0.655	0.958	0.797	0.896	0.387	0.884	0.326	0.925	0.562	0.940	0.665
		3	0.936	0.636	0.954	0.766	0.913	0.488	0.916	0.506	0.922	0.543	0.938	0.649
		4	0.927	0.575	0.956	0.778	0.941	0.673	0.909	0.461	0.922	0.543	0.938	0.652
		5	0.934	0.625	0.957	0.787	0.919	0.526	0.939	0.658	0.922	0.541	0.948	0.721
		6	0.936	0.639	0.962	0.819	0.824	0.126	0.888	0.347	0.931	0.606	0.948	0.726
		7	0.938	0.651	0.951	0.744	0.818	0.112	0.967	0.853	0.932	0.608	0.955	0.773
		8	0.935	0.63	0.956	0.778	0.934	0.621	0.971	0.884	0.927	0.576	0.955	0.773
		9	0.945	0.703	0.954	0.762	0.972	0.888	0.958	0.793	0.920	0.530	0.944	0.696
		10	0.946	0.707	0.948	0.720	0.951	0.745	0.954	0.768	0.925	0.563	0.946	0.705
	Levene statistic Sig.		1.000		1.000		0.999		0.998		1.000		1.000	
	Tail	1	0.984	0.955	0.987	0.969	0.912	0.479	0.898	0.399	0.912	0.479	0.957	0.79
		2	0.985	0.959	0.987	0.969	0.919	0.524	0.886	0.338	0.919	0.524	0.96	0.807
		3	0.989	0.977	0.982	0.945	0.92	0.53	0.914	0.491	0.92	0.53	0.959	0.798
		4	0.985	0.961	0.979	0.93	0.913	0.489	0.923	0.55	0.913	0.489	0.957	0.787
		5	0.984	0.955	0.989	0.974	0.909	0.464	0.9	0.412	0.909	0.464	0.953	0.756
		6	0.984	0.957	0.965	0.843	0.907	0.452	0.918	0.517	0.907	0.452	0.952	0.75
		7	0.988	0.973	0.984	0.954	0.905	0.438	0.925	0.566	0.904	0.434	0.963	0.826
		8	0.978	0.924	0.995	0.994	0.908	0.456	0.925	0.565	0.908	0.457	0.961	0.815
		9	0.974	0.899	0.994	0.991	0.927	0.578	0.93	0.597	0.927	0.578	0.954	0.767
		10	0.99	0.978	0.993	0.988	0.928	0.582	0.922	0.545	0.928	0.582	0.956	0.783
	Levene statistic Sig.		1.000		1.000		1.000		0.999		1.000		1.000	
Long-term	Head	10	0.946	0.707	0.92	0.528	0.979	0.93	0.931	0.601	0.919	0.52	0.955	0.772
		20	0.876	0.293	0.951	0.748	0.958	0.795	0.899	0.403	0.926	0.569	0.942	0.679
		30	0.847	0.187	0.924	0.555	0.976	0.912	0.907	0.45	0.944	0.694	0.936	0.637
	Levene statistic Sig.		0.970		0.998		0.976		0.741		0.959		0.982	
	Tail	10	0.996	0.997	0.973	0.897	0.892	0.367	0.942	0.681	0.892	0.367	0.947	0.713
		20	0.881	0.315	0.96	0.809	0.918	0.519	0.922	0.543	0.918	0.519	0.934	0.624
		30	0.993	0.99	0.956	0.779	0.915	0.498	0.918	0.516	0.915	0.498	0.946	0.711
	Levene statistic Sig.		0.885		0.985		0.969		0.651		0.969		0.974	
Abbreviations: CAT, catalase activity; SOD, superoxide dismutase activity; POD, peroxidase activity; MDA, malondialdehyde content; GST, glutathione S-transferase activity; GPX, glutathione peroxidase activity.														

## Appendix C. Analysis of Variance of Enzyme Activity in Earthworms under Oxytetracycline Stress

**Groups**	**Tissue**	**Indices**	**Exposure Time**					**Exposure Concentration**				
			**III-Sums of Squares**	**df**	**Mean Square**	**F Value**	**Sig.**	**III-Sums of Squares**	**df**	**Mean Square**	**F Value**	**Sig.**
Short-term	Head	CAT	276.291	9	30.699	71.575	0	8006.848	4	2001.712	4667.032	0
		SOD	88.5	9	9.833	12.906	0	24,969.136	4	6242.284	8192.754	0
		POD	67.193	9	7.466	2.075	0.059	2890.796	4	722.699	200.86	0
		MDA	18.582	9	2.065	0.805	0.614	4775.662	4	1193.915	465.762	0
		GST	64.359	9	7.151	66.613	0	8033.661	4	2008.415	18,708.515	0
		GPX	77.665	9	8.629	58.385	0	8727.362	4	2181.841	14,761.86	0
	Tail	CAT	52.925	9	5.881	9.017	0	5575.229	4	1393.807	2137.276	0
		SOD	81.531	9	9.059	4.776	0	14,755.069	4	3688.767	1944.855	0
		POD	72.12	9	8.013	83.078	0	6009.643	4	1502.411	15,576.324	0
		MDA	1.421	9	0.158	0.173	0.996	4604.688	4	1151.172	1259.084	0
		GST	71.396	9	7.933	72.262	0	5992.903	4	1498.226	13,647.709	0
		GPX	63.105	9	7.012	85.863	0	5926.588	4	1481.647	18,143.678	0
Long-term	Head	CAT	233.639	2	116.819	54.512	0	2074.226	4	518.557	241.975	0
		SOD	38.37	2	19.185	5.291	0.034	7733.931	4	1933.483	533.269	0
		POD	15.618	2	7.809	4.042	0.061	1666.204	4	416.551	215.579	0
		MDA	8.807	2	4.404	0.804	0.481	2827.165	4	706.791	128.969	0
		GST	81.381	2	40.69	51.058	0	2108.08	4	527.02	661.302	0
		GPX	111.788	2	55.894	118.74	0	2449.679	4	612.42	1301.009	0
	Tail	CAT	112.099	2	56.049	9.15	0.009	1369.05	4	342.263	55.874	0
		SOD	51.341	2	25.671	22.514	0.001	5888.623	4	1472.156	1291.149	0
		POD	55.665	2	27.833	61.302	0	2082.227	4	520.557	1146.533	0
		MDA	4.862	2	2.431	0.399	0.684	2429.834	4	607.458	99.598	0
		GST	55.617	2	27.809	61.235	0	2082.586	4	520.646	1146.479	0
		GPX	105.175	2	52.587	121.865	0	2228.599	4	557.15	1291.128	0
Abbreviations: CAT, catalase activity; SOD, superoxide dismutase activity; POD, peroxidase activity; MDA, malondialdehyde content; GST, glutathione S-transferase activity; GPX, glutathione peroxidase activity.												

## Appendix D. The Concentration of Oxytetracycline Concentration Detected by High-Performance Liquid Chromatography

**Experimental Setting Concentration** **(mg/kg)**	**Oxytetracycline Concentration in Soil** **(mg/kg)**	**Oxytetracycline Concentration in Earthworm** **(mg/kg)**
0.036	0.03	<0.01
0.36	0.32	
3.6	3.19	
36	32.58	
72	65.52	

## Appendix E. EC50 Data of *Q*

**Groups**	**Exposure** **Time (day)**	**EC 50 of *Q* (mg/kg)**	
		**Head**	**Tail**
Short-term	1	23.510	81.953
	2	24.719	107.282
	3	24.561	124.815
	4	20.778	105.317
	5	22.753	76.976
	6	21.503	94.562
	7	20.330	92.127
	8	22.602	88.409
	9	22.420	75.166
	10	22.498	65.412
Long-term	10	22.983	67.360
	20	22.620	66.252
	30	20.387	51.661

## References

[1] Li X., Xie Y., Wang J., Christakos G., Si J., Zhao H., Ding Y., Li J. (2013). Influence of planting patterns on fluoroquinolone residues in the soil of an intensive vegetable cultivation area in northern China. Sci. Total Environ..

[2] Wang R., Wei Y.S. (2013). Pollution and control of tetracyclines and heavy metals residues in animal manure. J. Agro-Environ. Sci..

[3] Barnes K.K., Kolpin D.W., Furlong E.T., Zaugg S.D., Meyer M.T., Barber L.B. (2008). A national reconnaissance of pharmaceuticals and other organic wastewater contaminants in the United States-I Groundwater. Sci. Total Environ..

[4] Kolpin D.W., Furlong E.T., Meyer M.T., Thurman E.M., Zaugg S.D., Barber L.B., Buxton H.T. (2002). Pharmaceuticals hormones and other organic wastewater contaminants in U.S. streams 1999–2000: A national reconnaissance. Environ. Sci. Technol..

[5] Scott G.I., Porter D.E., Norman R.S., Scott C.H., Uyaguari-Diaz M.I., Maruya K.A., Weisberg S.B., Fulton M.H., Wirth E.F., Moore J. (2016). Antibiotics as CECs: An overview of the hazards posed by antibiotics and antibiotic resistance. Front. Mar. Sci..

[6] Ya L., Liping Y., Dan L., Yunquan L., Ying P. (2023). Current situation of antibiotic contamination in China and the effect on plankton. Chin. J. Appl. Ecol..

[7] Wan Y.C., Li S.F., Suo Q.Y., Wen Z.X., Li M.Q. (2023). Effects of oxytetracycline contaminated organic fertilizer on soil bacterial diversity and structure. Res. Agric. Mod..

[8] Wang X., Zou C., Zhang Y., Shi X., Liu J., Fan S., Liu Y., Du Y., Zhao Q., Tan Y. (2018). Environmental impacts of pepper (*Capsicum annuum* L.) productionafected by nutrient management: A case study in southwest China. J. Clean. Prod..

[9] Dalorima T., Sakimin S.Z., Shah R.M. (2021). Utilization of organic fertilisers a potential approaches for agronomic crops: A review. Plant Sci. Today.

[10] Pan Z., Yang S., Zhao L., Li X., Weng L., Sun Y., Li Y. (2021). Temporal and spatial variability of antibiotics in agricultural soils from Huang-Huai-Hai Plain, northern China. Chemosphere.

[11] Li Y.W., Wu X.L., Mo C.H., Tai Y.P., Huang X.P., Xiang L. (2011). lnvestigation of sulfonamide, tetracycline, and quinolone antibiotics in vegetable farmland soil in the Pearl River Delta area, Southern China. J. Agric. Food Chem..

[12] Huang J., Yang T.A., Li Z.L., Zhou X.T., He Z.Q. (2022). Effects of different concentrations of tetracycline and oxytetracycline on the growth and ecotoxicity in lettuce. Chin. J. Appl. Environ..

[13] Zizek S., Zidar P. (2013). Toxicity of the ionophore antibiotic lasalocid to soil-dwelling invertebrates: Avoidance tests in comparison to classic sublethal tests. Chemosphere.

[14] Qu M., Xu Y., Chen H., Li Z., Sun L., Xu D., Kong Z., Sugiura N. (2005). Toxicological study of three veterinary drugs on Eisenia foetida. Chin. J. Appl. Ecol..

[15] Liu S., Tu X., Chen X., Mo L., Liu Y., Xu J., Deng M., Wu Y. (2023). Effects of single and combined exposure to zinc and two tetracycline antibiotics on zebrafish at the early stage. Comp. Biochem. Physiol. Part C Toxicol. Pharmacol..

[16] Fu R., Gong J., Zhang Q.Q. (2016). Effects of oxytetracycline on population growth and genetic diversity of euplotesvannus (marine protist). Sci. Sin..

[17] Kong W.D., Zhu Y.G., Liang Y.C., Zhang J., Smith F.A., Yang M. (2007). Uptake of oxytetracycline and its phytotoxicity to alfalfa (*Medicago sativa* L.). Environ. Pollut..

[18] Ahmed M.B.M., Rajapaksha A.U., Lim J.E., Vu N.T., Kim I.S., Kang H.M., Lee S.S., Ok Y.S. (2015). Distribution and accumulative pattern of tetracyclines and sulfonamides in edible vegetables of cucumber, tomato, and lettuce. J. Agric. Food Chem..

[19] Chi S.L., Wang W.Z., Xu W.H., Li T., Li Y.H., Zhang C.L. (2018). Effects of tetracycline antibiotics on growth and characteristics of enrichment and transformation in two vegetables. Environ. Sci..

[20] Bacanli M., Basaran N. (2019). Importance of antibiotic residues in animal food. Food Chem. Toxicol..

[21] Guo H., Xue S., Nasir M., Gu J., Lv J. (2021). Impacts of cadmium addition on the alteration of microbial community and transport of antibiotic resistance genes in oxytetracycline contaminated soil. J. Environ. Sci..

[22] Li M., Ma X., Saleem M., Wang X., Sun L., Yang Y., Zhang Q. (2020). Biochemical response, histopathological change and DNA damage in earthworm (*Eisenia fetida*) exposed to sulfentrazone herbicide. Ecol. Indic..

[23] Blouin M., Hodson M.E., Delgado E.A., Baker G., Brussaard L., Butt K.R., Dai J., Dendooven L., Peres G., Tondoh J.E. (2013). A review of earthworm impact on soil function and ecosystem services. Eur. J. Soil Sci..

[24] Zhou D., Ning Y., Wang B., Wang G., Su Y., Li L., Wang Y. (2016). Study on the influential factors of Cd^2+^ on the earthworm *Eisenia fetida* in oxidative stress based on factor analysis approach. Chemosphere.

[25] Ning Y., Wang S., Sun Y., Zhang S., Wen Y., Zou D., Zhou D. (2024). Deciphering survival strategies: Oxidative stress and microbial interplay in Eisenia fetida under tetracycline contamination. Sci. Total Environ..

[26] Lam P.K.S., Gray J.S. (2003). The use of biomarkers in environmental monitoring programmes. Mar. Pollut. Bull..

[27] Liang X., Zhou D., Wang J., Li Y., Liu Y., Ning Y. (2022). Evaluation of the toxicity effects of microplastics and cadmium on earthworms. Sci. Total Environ..

[28] Sanchez W., Burgeot T., Porcher J.M. (2013). A novel ‘integrated biomarker response’ calculation based on reference deviation concept. Environ. Sci. Pollut. Res..

[29] Wang K., Qiao Y., Li H., Huang C. (2020). Use of integrated biomarker response for studying the resistance strategy of the earthworm Metaphire californica in Cd-contaminated field soils in Hunan Province, South China. Environ. Pollut..

[30] OECD (2004). Guideline for Testing of Chemicals No. 222. Earthworm Reproduction Test (Eisenia fetida/Eisenia andrei).

[31] Ning Y., Li Y., Li X., Shao Z., Fu H., Yuan Y., Zhou D. (2022). Evolution of the earthworm (*Eisenia fetida*) microbial community in vitro and in vivo under tetracycline stress. Ecotoxicol. Environ. Saf..

[32] Wang X.D., Liu J., Wang G.N., Zhang D.X., Liu J.X. (2016). Residue Detection of tetracyclines in eggs by high performance liquid chromatography. China Anim. Husb. Vet. Med..

[33] Bradford M.M., Williams W.L. (1976). New, rapid, sensitive method for protein determination. Fed. Proc..

[34] Marklund S., Marklund G. (1974). Involvement of the superoxide anion radical in the autoxidation of pyrogallol and a convenient assay for superoxide dismutase. Eur. J. Biochem..

[35] Song Y., Zhu L., Wang J., Liu W., Xie H. (2009). DNA damage and effects on antioxidative enzymes in earthworm (*Eisenia foetida*) induced by atrazine. Soil Biol. Biochem..

[36] Goth L. (1991). A simple method for determination of serum catalase activity and revision of reference range. Clin. Chim. Acta.

[37] Flohe L., Günzler W.A. (1984). Assays of glutathione peroxidase. Methods Enzym..

[38] Habig W.H., Pabst M.J., Jakoby W.B. (1974). Glutathione S-transferases the first enzymatic step in mercapturic acid formation. J. Biol. Chem..

[39] Habig W.H., Jakoby W.B. (1981). Assays for differentiation of glutathione S-transferases. Methods Enzymol..

[40] Ohkawa H., Ohishi N., Yagi K. (1979). Assay for lipid peroxides in animal tissues by thiobarbituric acid reaction. Anal. Biochem..

[41] Hagger J.A., Jones M.B., Lowe D., Leonard D.P., Owen R., Galloway T.S. (2008). Application of biomarkers for improving risk assessments of chemicals under the Water Framework Directive: A case study. Mar. Pollut. Bull..

[42] Piva F., Ciaprini F., Onorati F., Benedetti M., Fattorini D., Ausili A., Regoli F. (2011). Assessing sediment hazard through a weight of evidence approach with bioindicator organisms: A practical model to elaborate data from sediment chemistry, bioavailability, biomarkers and ecotoxicological bioassays. Chemosphere.

[43] Beliaeff B., Burgeot T. (2002). Integrated biomarker response: A useful tool for ecological risk assessment. Environ. Toxicol. Chem..

[44] Zhou D., Liang X., Wang J., Wang S., Li X., Ning Y. (2021). Study on the regulatory mechanism of the earthworm microbial community in vitro and in vivo under cadmium stress. Environ. Pollut..

[45] Zhou H., Zhang T., Zhuang J., Xu M., Liu X., Shi Q., Zhou D. (2020). Study on the regulation of earthworm physiological function under cadmium stress based on a compound mathematical model. Environ. Toxicol. Pharmacol..

[46] Ning Y., Wang X., Lu J., Li Y., Yang Y., Zou D., Zhou D. (2023). Study on the life maintenance mechanism of *Eisenia fetida* under low-density polyethylene stress: Based on path analysis and canonical correlation analysis. Ecotoxicol. Environ. Saf..

[47] Zhou D., Wang S., Liang X., Wang J., Zhu X., Ning Y. (2020). The relationship between the oxidative stress reaction and the microbial community by a combinative method of PA and CCA. Sci. Total Environ..

[48] Cheng Y., Zhu L., Song W., Jiang C., Li B., Du Z., Wang J., Wang J., Li D., Zhang K. (2020). Combined effects of mulch film-derived microplastics and atrazine on oxidative stress and gene expression in earthworm (*Eisenia fetida*). Sci. Total Environ..

[49] Yu J.X., Wu C.Y. (2002). Application of biomarkers in rapid detection and toxicity evaluation of pollutants. Chin. J. Anal. Chem..

[50] Zhou C., Li C.H. (2007). Macromolecule biomarker detection used in environmental monitoring. J. Fish. Sci. China.

[51] Fanping M., Fenglian C., Jianchun W., Xiuping D., Zhengyan L., Yongfu L., You Z., Zhifeng W. (2013). Organic contamination assessment of Beibu Gulf intertidal zone with IBR index based on biomarkers of oxidative stress. Haiyang Xuebao.

[52] Colacevich A., Sierra M.J., Borghini F., Millán R., Sanchez-Hernandez J.C. (2011). Oxidative stress in earthworms short- and long-term exposed to highly Hg-contaminated soils. J. Hazard. Mater..

[53] Dongxing Z., Yucui N., Jiabin L., Jie D., Guohua R., Bilige S., Yijun L. (2016). Effects of oxidative stress reaction for the *Eisenia fetida* with exposure in Cd^2+^. Environ. Sci. Pollut. Res..

[54] Yang X., Song Y., Ackland M.L., Liu Y., Cao X. (2012). Biochemical responses of earthworm *Eisenia fetida* exposed to cadmium-contaminated soil with long duration. Bull. Environ. Contam. Toxicol..

[55] Lagesson A., Fahlman J., Brodin T., Fick J., Jonsson M., Byström P., Klaminder J. (2016). Bioaccumulation of five pharmaceuticals at multiple trophic levels in an aquatic food web-Insights from a field experiment. Sci. Total Environ..

[56] Jürgens M.D., Crosse J., Hamilton P.B., Johnson A.C., Jones K.C. (2016). The long shadow of our chemical past—High DDT concentrations in fish near a former agrochemicals factory in England. Chemosphere.

[57] Xu B., Janson J.C., Sellos D. (2001). Cloning and sequencing of a molluscan endo-β-1, 4-glucanase gene from the blue mussel, Mytilus edulis. Eur. J. Biochem..

[58] Bernard F., Brulle F., Douay F., Lemière S., Demuynck S., Vandenbulcke F. (2010). Metallic trace element body burdens and gene expression analysis of biomarker candidates in Eisenia fetida, using an “exposure/depuration” experimental scheme with field soils. Ecotoxicol. Environ. Saf..

[59] Peng L.S., Guan M.X., Huang K., Xia H., Sang C.L. (2022). Effects of excess sludge fed by earthworms on microbial community and antibiotic resistance genes in their intestinal functional area. China Environ. Sci..

[60] Bernard L., Chapuis-Lardy L., Razafimbelo T., Razafindrakoto M., Pablo A.-L., Legname E., Poulain J., Brüls T., O'Donohue M., Brauman A. (2012). Endogeic earthworms shape bacterial functional communities and affect organic matter mineralization in a tropical soil. ISME J..

[61] Guhra T., Stolze K., Schweizer S., Totsche K.U. (2020). Earthworm mucus contributes to the formation of organo-mineral associations in soil. Soil Biol. Biochem..

[62] (2008). Soil Quality—Avoidance Test for Determining The Quality of Soils and Effects of Chemicals on Behaviour—Part 1: Test with Earthworms (Eisenia fetida and Eisenia andrei).

[63] Yucui N., Haoran Z., Enze W., Congmin J., Ying Y., Xu C., Dongxing Z. (2021). Study of oxidative stress cadmium (Cd)-induced in *Eisenia fetida* based on mathematical modelling. Pedosphere.

[64] Ning Y., Liu L., Rong G., Cao X., Li J., Su Y., Zhou D. (2018). Study on the influential biochemical indices of Cd(II) on *Eisenia fetida* in oxidative stress by principal component analysis in the natural soil. Environ. Sci. Pollut. Res..

[65] Ning Y., Jin C., Zhou H., Wang E., Huang X., Zhou D. (2018). Screening indices for cadmium-contaminated soil using earthworm as bioindicator. Environ. Sci. Pollut. Res..

[66] Li X., Xing M., Yang J., Dai X. (2014). Earthworm eco-physiological characteristics and quantification of earthworm feeding in vermifiltration system for sewage sludge stabilization using stable isotopic natural abundance. J. Hazard. Mater..

[67] Palmer M.J., Moffat C., Saranzewa N., Harvey J., Wright G.A., Connolly C.N. (2013). Cholinergic pesticides cause mushroom body neuronal inactivation in honeybees. Nat. Commun..

[68] Zhu B.K., Fang Y.M., Zhu D., Christie P., Ke X., Zhu Y.G. (2018). Exposure to nanoplastics disturbs the gut microbiome in the soil oligochaete Enchytraeus crypticus. Environ. Pollut..

[69] Chao H., Kong L., Zhang H., Sun M., Ye M., Huang D., Zhang Z., Sun D., Zhang S., Yuan Y. (2019). *Metaphire guillelmi* gut as hospitable micro-environment for the potential transmission of antilbiotic resistance genes. Sci. Total Environ..

[70] Zhu D., Delgado-Baquerizo M., Su J.-Q., Ding J., Li H., Gillings M.R., Penuelas J., Zhu Y.-G. (2021). Deciphering potential roles of earthworms in mitigation of antibiotic resistance in the soils from diverse ecosystems. Environ. Sci. Technol..

[71] Hong S.W. (2011). Culture-based and denaturing gradient gel electrophoresis analysis of the bacterial community structure from the intestinal tracts of earthworms (*Eisenia fetida*). J. Microbiol. Biotechnol..

[72] Banerjee A., Biswas J.K., Pant D., Sarkar B., Chaudhuri P., Rai M., Meers E. (2019). *Enteric bacteria* from the earthworm (*Metaphire posthuma*) promote plant growth and remediate toxic trace elements. J. Environ. Manag..

[73] Wu Y.F., Ju J., Fu W.H., Xia S.Q., Wei J., Sun P.P., Zhang K.M., Zhao H.T., Feng K. (2019). Effect of earthworm swallowing on the tetracycline degradation and accumulation of macro-mineral element in sewage sludge. Chin. J. Environ. Eng..

[74] Cao J., Wang C., Dou Z., Liu M., Ji D. (2018). Hyphospheric impacts of earthworms and arbuscular mycorrhizal fungus on soil bacterial community to promote oxytetracycline degradation. J. Hazard. Mater..

[75] Dorval J., Hontela A. (2003). Role of glutathione redox cycle and catalase in defense against oxidative stress induced by endosulfan in adrenocortical cells of rainbow trout (*Oncorhynchus mykiss*). Toxicol. Appl. Pharmacol..

[76] Bonet B., Corcoll N., Guasch H. (2012). Antioxidant enzyme activities as biomarkers of Zn pollution in fluvial biofilms. Ecotoxicol. Environ. Saf..

[77] Sun S.H., Jiao C.Z., Liu X.L., Wei Z.L. (2009). Effects of cadmium (II) stress on xanthine oxidase and antionxidant enzyme activities in hepato pancreas of oriental weatherfish *Misgurnus anguillicaudatus*. J. Dalian Ocean Univ..

[78] Zhu S., Zhu L., Liu M., Chang C. (2010). Toxicity of commercial penta-BDE and Cd in soil on anti-oxidant defensive responses of earthworms. Environ. Sci. Technol..

[79] Zhou D.X., Wang X., Ning Y.C., Li J., Wu X.H., Wang G.D. (2017). Effect of oxidative stress reaction for *Eisenia fetida* with exposure in Cd. J. Northeast Agric. Univ..

[80] Wang Y., Tang Y., Xu L.-L., Diao X.-P. (2009). Ecotoxicolgical effects of albendazole on *Eisenia fetida*. Chin. J. Appl. Ecol..

[81] Bu Y.Q., Shan Z.J., Zhi Y., Zhang K., Jiang J.L. (2013). Physiological effect of exposure to nonylphenol polyethylene glycol ether on *Eisenia fetida*. J. Ecol. Rural Environ..

[82] Bai GuiFen B.G., Li Bing L.B., Huo ShuZheng H.S. (2014). Effect of carbendazim on activity of three detoxifying enzymes in earthworm. Guizhou Agric. Sci..

[83] Chi L., Gao B., Bian X., Tu P., Ru H., Lu K. (2017). Manganese-induced sex-specific gut microbiome perturbations in C57BL/6 mice. Toxicol. Appl. Pharmacol..

[84] Yang X., Gong J., Zhang X., Huang Y., Zhang W., Yang J., Lin J., Chai Y., Liu J. (2021). Evaluation of the combined toxicity of multi-walled carbon nanotubes and cadmium on earthworms in soil using multi-level biomarkers. Ecotoxicol. Environ. Saf..

[85] Alak G., Yeltekin A., Tas I.H., Ucar A., Parlak V., Topal A., Kocaman E.M., Atamanalp M. (2017). Investigation of 8-OHd G, CYP1A, HSP70 and transcriptional analyses of antioxidant defence system in liver tissues of rainbow trout exposed to eprinomectin. Fish Shellfish Immunol..

[86] Arslan H., Altun S., Ozdemir S. (2017). Acute toxication of deltamethrin results in activation of i NOS, 8-OHd G and up-regulation of caspase 3, i NOS gene expression in common carp (*Cyprinus carpio* L.). Aquat. Toxicol..

[87] Topal A., Alak G., Ozkaraca M., Yeltekin A.C., Comaklı S., Acıl G., Kokturk M., Atamanalp M. (2017). Neurotoxic responses in brain tissues of rainbow trout exposed to imidacloprid pesticide: Assessment of 8-hydroxy-2-deoxyguanosine activity, oxidative stress and acetylcholinesterase activity. Chemosphere.

